# Ego-Resiliency Reloaded: A Three-Component Model of General Resiliency

**DOI:** 10.1371/journal.pone.0120883

**Published:** 2015-03-27

**Authors:** Dávid Farkas, Gábor Orosz

**Affiliations:** 1 Institute of Cognitive Neuroscience and Psychology, Research Centre for Natural Sciences, Hungarian Academy of Sciences, Budapest, Hungary; 2 Department of Cognitive Science, Faculty of Natural Sciences, Budapest University of Technology and Economics, Budapest, Hungary; 3 Institute of Psychology, University of Eötvös Lóránd, Budapest, Hungary; 4 Institute of Psychology, University of Szeged, Szeged, Hungary; Middlesex University Dubai, UNITED ARAB EMIRATES

## Abstract

Ego-resiliency (ER) is a capacity that enables individuals to adapt to constantly changing environmental demands. The goal of our research was to identify components of Ego-resiliency, and to test the reliability and the structural and convergent validity of the refined version of the ER11 Ego-resiliency scale. In Study 1 we used a factor analytical approach to assess structural validity and to identify factors of Ego-resiliency. Comparing alternative factor-structures, a hierarchical model was chosen including three factors: Active Engagement with the World (AEW), Repertoire of Problem Solving Strategies (RPSS), and Integrated Performance under Stress (IPS). In Study 2, the convergent and divergent validity of the ER11 scale and its factors and their relationship with resilience were tested. The results suggested that resiliency is a double-faced construct, with one function to keep the personality system stable and intact, and the other function to adjust the personality system in an adaptive way to the dynamically changing environment. The stability function is represented by the RPSS and IPS components of ER. Their relationship pattern is similar to other constructs of resilience, e.g. the Revised Connor-Davidson Resilience Scale (R-CD-RISC). The flexibility function is represented by the unit of RPSS and AEW components. In Study 3 we tested ER11 on a Hungarian online representative sample and integrated the results in a model of general resiliency. This framework allows us to grasp both the stability-focused and the plasticity-focused nature of resiliency.

## Introduction

Block [1] conceptualized personality as an affect processing system in which *ego-resiliency* (ER) is coupled with *ego-control* (EC). These two constructs constitute the foundations of Block’s theory of personality. In this system, EC is responsible for controlling one’s impulses in specific situations (including, for example, expression of aggression, spontaneity, and inhibition). ER then functions as a dynamic capacity which systematically modifies control, optimizing the personality system with regards to the environmental context [2]. In other words, EC is a meta-dimension of impulse inhibition and expression, whereas ER allows temporal and adaptive shifts between different degrees of control [3].

Block based his concept on Lewin’s [4,5] theory about the functioning of the psychological system of the individual. One of the central concepts in Lewin’s work is elasticity, which enables the psychological system to change between different degrees of permeability. Permeability is responsible for controlling the information change between subsystems, and it was incorporated into Block’s [1] theory as ego-control. Lewin’s elasticity is similar in function to ER, because ER enables the system to change control dynamically, as a result of interactions between the environment and the individual.

One of the most important contributions of Block and Block [6] is that by reinterpreting Lewin’s notions of elasticity and permeability they crystallized the concept of ER and made it measurable. Lewin [4,5] described the mechanisms of the psychological system in terms of elasticity and permeability, which was complemented by Block and Block [6] who measured individual differences in these processes in terms of ER and EC, and made them the core of personality. The concept of ER encompasses traits that emphasize flexibility and resiliency toward constantly varying situations and a general resourcefulness of personality [6]. This is useful behaviorally: ego-resilient individuals are intelligent, resourceful and adaptive in stressful situations [7]. For example, ego-resilient individuals tend to find more positive meaning in a problem compared to ego-brittle ones [8], and conversely, the presence of attachment-related anxiety, rumination and negative affect predicts lower rates of ER [9]. Ego-resilient individuals are ambitious, extraverted [10], and less likely to use self-enhancement [11]. ER is highly correlated with the delay of gratification, while EC is not [12].

ER has good temporal stability according to a 30-year-long longitudinal study [13]. A genetic basis for ER was identified in children aged from 18 to 84 months: the S10 haplotype that combines information from two genetic variants of the serotonin transporter gene was associated with ER [14]. Socialization also plays an important role in the development of ER. The quality of parenting, such as maternal sensitivity, intrusiveness, warmth, and verbal control combined with the genetic factors has an effect on the level of ER [14]. At the age of four, children who were more socially competent were found to be more ego-resilient [15]. Prosocial behavior of 6 and 7 years old children was predicted by the development of empathy, which was predicted by ER and other parenting measures [16]. During high school and college, ER was associated with higher social support, and ego-resilient individuals were associated with less internalizing symptoms [17]. ER is associated with a “flexible invocation of the available repertoire of problem solving strategies (problem-solving being defined to include the social and personal domains as well as the cognitive)”, an “integrated performance while under stress” and an “active engagement with the world but not subservient of it” [6] (p .48). In sum, ER both develops through and promotes socialization.

The scale measuring ER (ER89) was developed over 30 years ago, and some sources are unfortunately untraceable [2]. Moreover, the ER89 scale’s validity which has been used in the last 20 years to measure ER was only tested with factor analysis by Alessandri and Vecchione et al. [18–20] in its original form, and their results did not support the original structure. Therefore, the main goal of this study was to find meaningful internal structure in the ER meta-trait by taking into account Block’s [1] theoretical assumptions. In doing this, we assumed that different aspects of ER could function independently of each other under certain circumstances. In other words, we intended empirically to identify the components of ER—which were merged in the theory of Block and Block [6]—in order to establish a multidimensional measurement of adaptive flexibility.

The notion of “resiliency” was conceptualized in the 1950s and first reported by Block [21] in a psychological context, and led to a great deal of fruitful research during the nineteen seventies on resilience. Initially the concept was applied to children where it was known as “invulnerability” or “stress-resistance”; youngsters who did not have any psychopathology despite very difficult childhood circumstances were thought to be characterized by this trait. Later this research encompassed many different models, and nowadays resilience is understood as a stress-protective and health promoting factor, which contributes to well-being and good quality of life [22], as well as psychological growth and development [23].

While both Block’s ER and resilience are treated as protective factors against adversity, they are different in many ways. On the one hand, resilience presupposes exposure to substantial adversity and is interpreted as a dynamic process rather than a personality trait [24]. On the other hand, ER can be understood within his theory of personality, combined with EC. This theory-specific nature of ER might be one of the reasons why it is less used or even forgotten in current research; for example, Richardson’s [23] review on resilience fails to mention Block’s conceptualization of ER.

We aimed to study flexibility in a general way. Although Block’s conceptualization is specific to a certain theory, the basic ideas behind ER are more general than those behind resilience, as Block’s theory tries to describe the functioning of the whole personality, while resilience is concerned with only one aspect of it. In this study we chose Block’s conceptualization and forty years of work on ego-resiliency [21,13] as our theoretical and methodological cornerstone. We agree with Block’s interpretation of ego-resiliency as a meta-trait, because it reflects the flexibility of personality as a higher order organization. This notion of resiliency is activated in a wider range of situations than is conceived by the more recent theories of resilience. We intended to explore how Block’s approach to ER relates to the more recent resilience measures. Therefore, beyond the exploration of the factor structure of the ER scale, we aimed to shed light on potential advantages and disadvantages of Block’s ER scale, compared to more recent resilience measures, such as the Connor-Davidson Resilience Scale [25]. Our ultimate aim was to provide a measure of general flexibility which can be interpreted on its own without the complex theoretical background of Block.

### Overview of the Present Studies

In a series of studies, we tried to identify the components of ER and test the validity and reliability of the refined scale. In Study 1 we used a factor analytical approach to assess the structural validity of ER and to identify its facets. In Study 2 we tested the convergent and the divergent validity of the components by linear regression methods. Finally, in Study 3 we tested ER on a Hungarian online representative sample, to develop standards for applied research.

## Study 1

Block [1] defined ER along a continuum: ego-resilient persons have the flexibility to adapt to changing contexts and to coordinate their behavior with situational demands and behavioral possibilities. In contrast, ego-brittle individuals cannot resourcefully respond to the dynamically changing requirements of the environment and they tend to perseverate. Our goal was to identify through factor analytical methods the various aspects of ER underlying these capabilities.

For many years, ER was measured by the California Q-sort inventory [26], but later the ER89 scale was developed and used [2]. This scale measures ER as a one-dimensional construct. However, some studies suggest a different factor structure of the scale. Khlonen [27] added items from the California Personality Inventory [28], and discovered four factors of ER through exploratory and confirmatory factor analysis, which she named (a) Confident Optimism, (b) Productive Activity, (c) Insight and Warmth, and (d) Skilled Expressiveness. In a series of studies, Alessandri and Vecchione analyzed and tested the ER89 with their colleagues [18–20]. In their hierarchical model, ER was defined by two first-order factors, namely Openness to Life Experiences and Optimal Regulation. This new scale (i.e. ER89-R) measures ER as an indicator of positive social functioning and resilience.

In the present study we aimed to test the structural validity of the original ER89 scale on a Hungarian sample[2]. We also intended to test the factor structure previously identified by Alessandri, Vecchione, and their colleagues [18–20] using confirmatory factor analysis and exploratory structural equation modeling and to explore alternative factor structures, rooted in Block’s original concept of ER.

### Methods

#### Participants and measures

For the factor analyses, 1473 respondents (68.0% female), aged between 18 and 78 years (*M* = 26.3, *SD* = 9.5) filled out the Hungarian online version of the ER89 scale [29] and provided their demographic data (i.e. gender, age, education). The ER89 is a 14-item scale, where subjects have to indicate how much the items apply to them on a four-level Likert-scale from “Does not apply at all” to “Applies very strongly”. Previously, ER89 had a Cronbach’s alpha value between .73 and .81 [20,9,2]. ER89 is included in S1 File. Data was collected in two waves (in January 2012 and in March 2013). Participants were informed about the details of the study and were informed that by completing the questionnaire they consented to the use of their answers in our research. Voluntary response was emphasized in the instructions and anonymity was assured. Data collection was conducted in accordance with the Helsinki declaration, and was approved by the United Ethical Review Committee for Research in Psychology (EPKEB). Underage responses were not included in the analysis, and they will not be publicly available.

We wanted to avoid overfitting of the models. Overfitting refers to a problem that usually occurs when a model is trained on one dataset. In this case the model might explain that specific data very well, while it lacks exploratory or predictive value on other data. One way to overcome this problem is to use a validation approach [30]. In this type of analysis the data is split in half: one half is used to create a model, while the other is used to test the model. The rationale is that if the model predicts data other than that used to train it, then it is more likely to have general validity. The ER89 scale has most often been tested in adolescents [2] and undergraduate university students [3]. For compatibility with these investigations and to have more homogeneous datasets in the cross-validation approach, we first selected the age group of young adults between 20 and 30 years of age. This group covered 73.4% of our original sample (*N* = 1080; 67.7% female; *M*_age_ = 23.03, *SD*_age_ = .47). The sample was split into two halves by randomly assigning each respondent either to the Training Sample (*N* = 540, 66.3% female, *M*_age_ = 23.02, *SD*_age_ = 2.55) or the Test Sample (*N* = 540, 69.2% female, *M*_age_ = 23.03, *SD*_age_ = 2.56). The Training Sample was used for establishing an appropriate factor structure of the ER89 scale, while the Test Sample was used for testing and confirming the alternative models. In this selection almost 400 respondents were left out (*N* = 393, 68.7% female), aged 18 to 78 (*M* = 35.28, *SD* = 14.5). We used this data in a second validation of the best fitting models. In addition, test-retest reliabilities of the subscales were established in 91 respondents (73.1% female, age ranged between 20 and 30 years, *M* = 23.78, *SD* = 3.16) over a four week period. The data can be accessed from http://real.mtak.hu/10687/.

#### Data analysis

Confirmatory factor analysis (CFA) and Exploratory Structural Equation Modeling (ESEM) [31] were performed with Mplus 6.1. With the exception of item 7 (kurtosis _all participants_ = -1.07, kurtosis _training sample_ = -1.23, kurtosis _test sample_ = -1.06), all variables were normally distributed based on their skewness and kurtosis. Because variables are ordered categorical, the Mean- and Variance-adjusted Weighted Least Squares (WLSMV) estimator of Mplus 6.1 was used. This estimator is also robust for the non-normality of item 7. In the ESEM the oblique geomin rotation was used. There were no missing data in the scales. Following Tabachnik and Fidell’s [32] guidelines, the minimum loading of an item was .32 and cross-loading was interpreted as if an item was loaded at .32 or higher on two or more factors.

In the CFA analyses higher order and bifactor models [33] were also tested. Following the guidelines of Brown [34] and Schreiber, Stage, King, Nora and Barlow [35] several different indices of goodness of fit were taken into consideration, including chi-square degree of freedom ratio (χ^2^/df), root mean square error of approximation (RMSEA), comparative fit index (CFI), and the Tucker–Lewis index (TLI). Guided by the suggestions of Hu and Bentler [36], and Kline [37], acceptable model fit was defined by the following criteria: RMSEA (≤ .06,), CFI (≥ .95), and TLI (≥ .95). In contrast to CFA, ESEM allows exploratory testing of alternative factor structures, and it allows cross-loadings of items similar to exploratory factor analysis (EFA). Its main advantage compared to classical EFA is that the same model fit indices provided by the CFA are also provided by the ESEM. Thus, a direct comparison of exploratory and confirmatory models is possible. Furthermore, it provides a more efficient way to identify the appropriate number of factors than classical methods based on eigenvalues [31]. Internal consistency of the scales was assessed by Cronbach’s alpha using the item intercorrelation matrix.

We also used a model-based reliability measure, omega [33], which shows the variance of latent constructs relative to the overall observed score variance. In case of higher-order and nested models, omega measures the amount of variance explained by a factor to the specific and the overall constructs. Reliability in this method can be defined as variance accounted for specific constructs by one factor relatively to the overall observed variance in the model. This latter measure is called omega hierarchical. We reported omega and omega hierarchical besides alphas, because higher-order and bifactor models alphas cannot accurately capture the reliability of the different levels (general vs. specific factors) of the model [33,38].

### Results

The results of the analyses are summarized in Table 1. First, we tested the original, one factor structure that had been proposed for the ER89 [2] by CFA on the Training Sample (Model 1). While the scale had acceptable internal consistency and reliability, CFA showed poor model fit. We also tested the proposed structure of the ER89-R (Model 2), but the model fit was not satisfactory. The Optimal Regulation dimension showed poor reliability, while the Openness to Life Experiences and the higher order ER factor showed relatively weak or borderline internal consistency.

**Table 1:** Exploratory SEM and confirmatory factor analysis of the ego-resiliency scale.

Model	No. of items	α	ω	ω_h_	χ^2^	χ^2^/DF	CFI	TLI	RMSEA
*Training Sample*									
1. ER89	ER = 14	ER = .723	ER = .842		442.42	5.75	.849	.821	.094
2. ER89-R	OL = 4	OL = .662	OL = .677	OL = .385	164.12	4.83	.880	.841	.084
	OR = 6	OR = .415	OR = .427	OR = .243					
		ER = .621	ER = .632	ER = .380					
3. Best ESEM	1 = 2	1 = .650			71.69	2.87	.979	.954	.059
	2 = 5	2 = .723							
	3 = 4	3 = .580							
		ER = .754							
4. First-order	1 = 2	1 = .650	1 = .670	1 = 2	107.62	2.62	.970	.960	.055
	2 = 5	2 = .723	2 = .742	2 = 5					
	3 = 4	3 = .580	3 = .586	3 = 4					
		ER = .754							
5. Hierarchical	1 = 2	1 = .650	1 = .670	1 = .526	107.62	2.62	.970	.960	.055
	2 = 5	2 = .723	2 = .742	2 = .216					
	3 = 4	3 = .580	3 = .586	3 = .242					
		ER = .754	ER = .801	ER = .636					
6. Bifactor				No convergence					
*Test Sample*									
7. ER89	ER = 14	ER = .728	ER = .845		469.30	6.09	.841	.812	.097
8. Hierarchical	1 = 2	1 = .648	1 = .692	1 = .482	115.05	2.81	.968	.958	.058
	2 = 5	2 = .662	2 = .684	2 = .073					
	3 = 4	3 = .680	3 = .682	3 = .344					
		ER = .768	ER = .814	ER = .678					
*Test Sample2*									
9. ER89	ER = 14	ER = .732	ER = .852		327.37	4.25	.846	.818	.091
10. Hierarchical	1 = 2	1 = .683	1 = .685	1 = .526	94.09	2.29	.965	.952	.057
	2 = 5	2 = .660	2 = .684	2 = .210					
	3 = 4	3 = .599	3 = .602	3 = .079					
		ER = .758	ER = .797	ER = .673					

*Notes*: α = Cronbach’s alpha, χ^2^ = Chi-square, χ^2^/DF = Chi-square / Degree of freedom ratio; CFI = comparative fit index, TLI = Tucker-Lewis index, RMSEA = root mean square of approximation.

**Fig 1:**
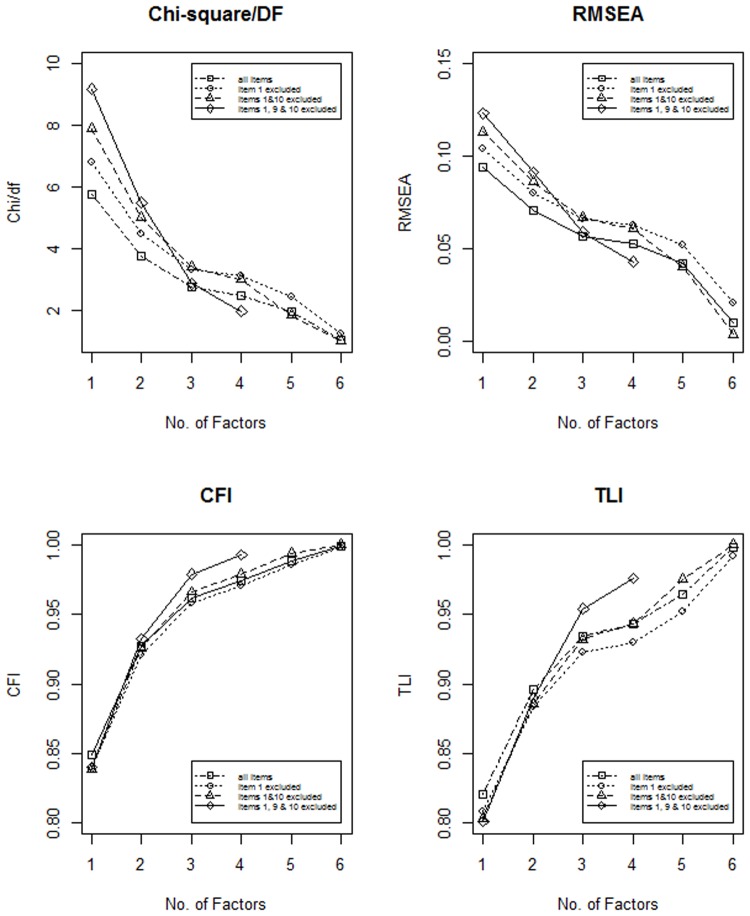
The fit indices of the ESEM models of ER. The X-axis is always the number of factors, while the Y-axis is a fit index. Upper left corner: Chi-square / degrees of freedom. Upper right corner: Root mean square error of approximation. Lower left corner: Comparative Fit Index. Lower right corner: Tucker-Lewis Index. Different types of lines mark the number of items included in the analysis (first item 1 was excluded, then item 10, then item 9). Factors did not converge after the exclusion of the three ill-fitting items above four solutions. The ‘elbow’ is visible at three factors in all cases.

In order to find an appropriate model that fitted our data we conducted an ESEM, which helps to define the number of factors and to assess model fit indices similarly to CFA. In all cases, a model based on three factors was favored (see Fig. 1). In three steps, items one, ten, and nine were excluded, because they did not load onto any of the best fitting three factors or just defined a factor on their own. Consequently, the best fitting model of this analysis was a three factor model based on eleven items (Model 3). For the fit indices of the models of ESEM see Fig. 1.

In Model 3, all cross-loadings were below .175. Thus, we tested this model using CFA (Model 4), which showed a very similar fit to the ESEM model. Beyond this first-order model a second-order one was also tested (Model 5), but adding a hierarchical ER latent variable did not improve model fit. The dimensions of the ER had internal consistencies ranging from borderline to acceptable in both models. A bifactor model (Model 6) was also tested, but it did not converge. The difference between the first-order and hierarchical models lies in their interpretation as well as in their fit. Therefore, we decided to test the hierarchical model on the test samples, because we propose that the relationship between ER and its dimensions can be grasped better by this structure compared to the first-order one.

**Fig 2:**
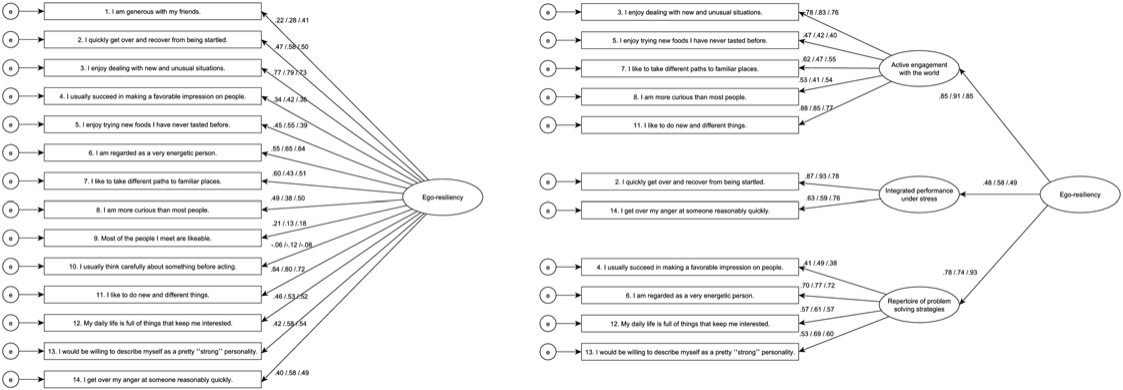
The CFA models of Ego-resiliency. Left side shows the original ER89 structure. Model 1: CFI = .849, RMSEA = .094, Model 7: CFI = .841, RMSEA = .097, Model 9: CFI = .846, RMSEA = .091. Right side shows the hierarchical ER11 structure. Model 4: CFI = .970, RMSEA = .055, Model 8: CFI = .968, RMSEA = .058, Model 10: CFI = .965, RMSEA = .057. One-headed arrows between the latent and observed variables show the standardized regression weights. The first value belongs to the Training Sample, after the slash the second value refers to the Test Sample. After the final slash the values of Test Sample 2 are noted.

The Test Sample was used to confirm the above mentioned factor structures. The proposed factor structure for the ER89 did not have a good fit (Model 7), but the hierarchical model (Model 8) had good model fit indices and acceptable reliabilities. Finally, we tested these models on the heterogeneous Test Sample 2. The models had similar fit indices and reliabilities as they did in the other two samples. Based on these results we accepted the hierarchical three-factored structure (Model 5, 8, and 10) as the methodologically—best fitting—and the theoretically—based on Block’s meta-trait notions—most appropriate model. The original factor structure and the accepted hierarchical structure are visualized in Fig. 2 with factor loadings from all three samples.

We have given names to the three factors based on Block and Block’s [6] description of ER. The first factor contains items 3, 5, 7, 8 and 11, and can be described as *Active engagement with the world*. The second factor includes items 2 and 14 and it reflects *Integrated performance under stress*. The third factor consists of items 4, 6, 12, and 13 and captures the *Repertoire of* (cognitive, social and personal) *problem solving strategies*. The test-retest correlations after four weeks of the overall scales (ER89 and ER11) and the three subscales were high (see Table 2).

**Table 2:** Test-retest Pearson-correlations of the different versions and factors of the ego-resiliency scale.

	ER89	ER11	AEW	IPS	RPSS
Test-retest	.807	.844	.845	.636	.801

ER89 = Block & Kremen’s (1996) original version of the scale; ER11 = the present version of the ER; AEW = active engagement with the world; IPS = integrated performance under stress; RPSS = repertoire of problem solving strategies.

*Note*: N = 91, all significance levels were *p* < .001

### Discussion

Study 1 was conducted to test the one-factor and ER89-R models, and to find an acceptable factor structure for the shortened ER89 scale [2]. Results indicated an 11-item (ER11) hierarchical structure with three specific factors. It is important to note that there were no differences in model fit indices between the first-order and hierarchical models. The reason the hierarchical structure was preferred is based on theoretical considerations: the factors depict components of the ER meta-trait. The three specific factors are conceptualized on the basis of Block and Block’s [6] theory as the three components of ER. However, a limitation of this structure is that according to Cronbach’s alpha and the omegas, the internal consistency and the reliability of the three factors were borderline.

Although previously a two-factor solution was identified [18–20], the present results indicate a solution with one general ER factor, and three specific factors. This solution fits Block’s theoretical ideas well. Block and Block [6] described processes underlying ER as: (a) *Active engagement with the world* (AEW)–which indicates that an individual is continuously looking for new information and experiences in everyday events; (b) *Repertoire of (cognitive*, *social and personal) problem solving strategies* (RPSS)–which calls attention to the fact that adaptive flexibility can function only when it is backed up by the appropriate skills; and (c) *Integrated performance under stress* (IPS)–which describes the ability to recover quickly after stressful, unexpected events. It is worth mentioning, that AEW is nearly identical to Alessandri, Vecchione *et al*.’s [18–20] Openness to Life Experiences factor.

With these three aspects of ego-resiliency separated, it is possible to obtain more detailed information about an individual's resiliency. The results suggest that three potential subsystems of ER can be assumed: AEW might be responsible for the information uptake and selection through an open-minded information seeking tendency; RPSS might be required for the appropriate problem solving strategies, and IPS might be activated in stressful situations. The three factors constitute ego-resiliency as a meta-trait in an interrelated way. The test-retest correlations show that these constructs are temporally stable. We assume that this factor structure allows grasping a more general concept of flexibility and resiliency compared to other scales that were designed to assess more specific forms of resilience. This assumption will be tested in Study 2.

## Study 2

The validity of the ER89 scale has previously been tested primarily in relation to other scales [2,3]. Following this practice, Study 2 was conducted to assess the pattern of relationships of the three factors of ER with other personality constructs. The first aim of Study 2 was to compare ER11 to ER89 in terms of their relationship to other variables, in order to examine whether the exclusion of three items can lead to the same relationship patterns as the original scale. Our second aim was to check the validity and the relationship patterns of the dimensions of ER, and our third aim was to examine the discriminant validity of ER11 and resilience. To this end, beyond filling out the ER89 scale [2], respondents also filled out the refined Connor-Davis Resilience Scale (R-CD-RISC [25]). R-CD-RISC measures a specific type of resiliency focusing on positive adaptation to stress and trauma [24]. For identifying similarities and differences between the two forms of resiliency, their relationship pattern was compared considering several other relevant constructs.

First, both measures should indicate a higher quality of life, and they are supposed to be related to better coping with negative emotions. For measuring this aspect, the Spielberger Trait Anxiety Inventory (STAI [39]) was used to assess both state and trait anxiety. The Positive and Negative Affect Scale (PANAS, [40,41]) was applied to measure the current and the general state of positive and negative emotions. Participants filled out the Subjective Well-Being Scale [42] to measure the overall satisfaction with life. According to previous research, both ER and resilience were related negatively to negative emotional states, while they were positively related to positive emotions and subjective well-being [43,9,44–50].

Second, we measured the Big Five personality traits [51] with the Big Five Inventory [52] to connect these general and widely used traits to ER and resilience. According to previous results both ER [53,3] and CD-RISC [54,48] were related positively to every Big Five trait with the exception of Neuroticism. We took into account the work of DeYoung and his colleagues [55–58] who identified two meta-traits. The first meta-trait, Stability, is composed of Agreeableness, Conscientiousness, and Emotional stability. This meta-trait describes the need to maintain a stable functioning psychological system, and might be related to serotoninergic functioning. The other meta-trait, Plasticity, includes Extraversion and Openness. This meta-trait describes an individual’s tendency to explore and integrate new information and to integrate it without destabilization. This function might be related to the dopaminergic system [55,57]. These two meta-traits are in line with the elasticity (plasticity) and permeability (stability) notions of Lewin, and hence are also in accordance with Block’s notion of ER and ego-control.

A number of more specific constructs were included, as well. A measure of General Self-Efficacy [59] that describes the beliefs in one’s ability to mobilize one’s resources to accomplish a given task, which is related to resilience [60,61]. Resilience and creativity are connected [62], thus the Biographical Inventory of Creative Behaviors [63] was also included in the test battery. The scale converges with self-reports of creativity, and has been proved to be a valid measure of everyday creativity [64].

### Methods

#### Participants

Two different samples were used. Sample 1 consisted of 144 respondents (70.1% female) aged between 18 and 61 years (*M* = 28.24, *SD* = 8.31). Sample 2 consisted of 321 respondents (77.3% female) aged between 18 and 72 (*M* = 32.14, *SD* = 11.15). Data for Sample 1 was collected in January 2014, and for Sample 2 in July-August 2014. All participants were assured of their anonymity, and informed about the details of the study. They were told that by completing the questionnaire they consented to let us use their answers in our research. Data collection was conducted in accordance with the Helsinki declaration, and approved by United Ethical Review Committee for Research in Psychology (EPKEB). Underage responses could not be controlled before filling in the scales, but this data was not included in the analysis and will not be publicly available.

#### Measures and Procedure

Convergent validity of the ER11 scale and its subscales was investigated by using 7 questionnaires (17 scales) in two samples. Respondents of the two samples first responded to *demographic questions* (age, gender, degree obtained, place of living) and the *ER89* scale [2]. Respondents in Sample 1 filled out (a) the *refined CD-RISC* scale [25], which is a 10-item scale with one factor, scored on a 5-point Likert scale ranging from”Not true at all” to “True nearly all the time”; (b) the *trait anxiety* section of the *STAI* [39], which is a 20-item scale with one factor, scored in a 4-point Likert scale ranging from”Never” to “Often”; (c) the *Subjective Well-Being Scale*[42], which is a five-item, one-factor scale on a 7-point Likert scale ranging from”Strongly disagree” to “Strongly agree”; (d) a 15-item version of *BFI* [29], which measures the Big Five traits (Extraversion, Agreeableness, Conscientiousness, Emotional Stability, and Openness) with three items each. This was not included in the analysis, as participants in Sample 2 completed the original 44-item version of the scale. We did not want to report the Big Five traits twice, hence we choose to report the one which was completed by more participants. Furthermore, using the original 44-item version supports cross-cultural comparisons better than the short version. Finally, participants filled out (e) the *state anxiety* section of the *STAI*, which measures anxiety on 20-items and with one factor, scored on a 4-point Likert scale ranging from”Never” to “Often”. Data can be accessed from http://real.mtak.hu/10688/.

Respondents in Sample 2 filled out the (a) *PANAS* [40,41] scale, which is used to indicate the extent of feeling of positive and negative affects separately. It uses a 5-point scale ranging from”Very slightly or not at all” to ‘”Extremely”‘. Respondents were asked to indicate how they were feeling at the exact moment. Second, the (b) *ER89* and (c) *R-CD-RISC* scales were filled out. After that they filled out the (d) *Big Five Inventory* [52], which measures the big five traits, namely Extraversion (eight items), Agreeableness (nine items), Conscientiousness (nine items), Emotional stability (eight items), and Openness (ten items). Respondents rated the items on a 5-point scale ranging from”Strongly disagree” to “Strongly agree”. The dimensions of the Big Five Inventory were used to calculate the two meta-traits of DeYoung and his colleagues [55–58]: Extraversion and Openness for Plasticity, and Emotional Stability, Conscientiousness, and Agreeableness for Stability.

Next, the (e) *New General Self-Efficacy Scale* [59] was filled out. This measures general self-efficacy on eight items with a 5-point scale ranging from”Disagree strongly” to “Strongly agree”. Participants then filled out the (f) *PANAS* again, but in this case the instruction was to indicate their affective state on the basis of their last week's experiences in order to obtain data about their general affective state. Finally, respondents filled out the (g) *Biographical Inventory of Creative Behaviors* (BICB) [63] scale, which has 34 items describing everyday creative behaviors as “Organized an event” or “Wrote a poem”. Respondents indicated if they had executed any of these behaviors in the last 12 months with”yes” or “no”. The last two scales were not available in the Hungarian language so they were translated using the protocol of Beaton and colleagues [65].

#### Data analysis

Statistical analyses were performed using R and SPSS for Windows 15. Internal consistency was tested with Cronbach’s alpha. Convergent validity of ER89 and ER11 was tested based on their Pearson correlation coefficients with the other measured scales. The correlation coefficients were compared with Williams’ *t* [66], which is one of the most reliable tools for comparing correlation coefficients in dependent samples [67]. Incremental validity of ER11 and R-CD-RISC was tested with hierarchical multiple regressions. For each dependent variable two models were created: one, in which ER11 was included in the first step and R-CD-RISC was placed in the second step; and a second in which the order of the two resilience measures was reversed. Convergent and divergent validity of the dimensions of ER11 were tested with multiple linear regression for every other measured variable.

### Results

Table 3 depicts the descriptive results and internal consistencies of the different versions of the ER scale across the two samples. For a description of the other scales used see Table 4. All variables had a normal distribution based on their skewness and kurtosis.

**Table 3:** Descriptive Statistics and Internal Consistencies of Different Versions of Ego-resiliency Scales.

	Sample	Observed range	Number of items	Mean	SD	Alpha
ER89	Sample 1	1–4	14	2.89	.33	.68
	Sample 2			3.00	.41	.75
ER11	Sample 1	1–4	11	2.87	.37	.68
	Sample 2			2.97	.48	.77
AEW	Sample 1	1–4	5	2.87	.52	.67
	Sample 2			3.02	.59	.67
RPSS	Sample 1	1–4	4	2.97	.46	.57
	Sample 2			3.05	.60	.67
IPS	Sample 1	1–4	2	2.64	.71	.64
	Sample 2			2.67	.77	.74
R-CD-RISC	Sample 1	1–5	10	3.80	.59	.83
	Sample 2			3.80	.66	.85

*Note*: ER89 = Block & Kremen’s (1996) ego-resiliency scale, ER11 = our version of the ER scale, AEW = active engagement with the world, RPSS = repertoire of problem solving strategies, IPS = integrated performance under stress, R-CD-RISC = Refined Connor-Davidson Resiliency Scale

**Table 4:** Descriptive Statistics and Internal Consistencies of the Used Questionnaires and Their Subscales.

Sample 1	(N = 144)	Observed range	Number of items	Mean	SD	Alpha
STAI	Trait	1–4	20	2.17	.44	.90
	State	1–4	20	1.93	.60	.95
SWB		1–7	5	5.01	1.22	.86
Sample 2	(N = 321)	Observed range	Number of items	Mean	SD	Alpha
PANAS	Positive Affect (moment)	1–5	10	3.26	.77	.88
	Negative Affect (moment)	1–5	10	1.63	.68	.90
	Positive Affect (week)	1–5	10	3.63	.83	.91
	Negative Affect (week)	1–5	10	1.91	.80	.89
BFI	Extraversion	1–5	8	3.50	.75	.82
	Agreeableness	1–5	9	3.65	.65	.77
	Conscientiousness	1–5	9	3.61	.62	.78
	Emotional Stability	1–5	9	2.81	.73	.87
	Openness	1–5	10	3.90	.56	.78
General Self-Efficacy		1–5	8	4.13	.69	.92
BICB		0/1	34	9.43	5.08	.81

*Notes*: STAI = State and Trait Anxiety Inventory, SWB = Subjective Well-Being Scale, PANAS = Positive and Negative Affect Scale, BFI = Big Five Inventory, BICB = Biographical Inventory of Creative Behaviors

The inter-correlation matrix of Sample 1 including the results from the Williams’s *t*-test can be found in Table 5, and for Sample 2 it can be found in Table 6. Comparison of the correlation coefficients of ER11 and ER89 revealed that ER89 is significantly related more to R-CD-RISC and State Anxiety in Sample 1 than ER11. In Sample 2, ER89 was related significantly more to the Agreeableness and Conscientiousness traits, and to the Stability meta-trait of the Big Five than ER11. In both samples, ER11 was more related to the AEW component than the ER89.

**Table 5:** Inter-correlation matrix for Study 2–Sample 1 and comparison of the correlations of the ER11 and ER89 scales.

	1.	2.	3.	4.	5.	6.	7.	8.	Williams’ *t*
1. ER89									
2. ER11	.963***								
3. AEW	.745***	.792***							-3.36***
4. RPSS	.667***	.676***	.222**						-.45
5. IPS	.563***	.571***	.173*	.261***					-.63
6. R-CD-RISC	.642***	.603***	.284***	.576***	.485***				2.23*
7. Trait Anxiety	-.539***	-.500***	-.118	-.535***	-.541***	-.683***			-1.99*
8. State Anxiety	-.310***	-.278***	-.023	-.335***	-.330***	-.494***	.737***		-1.47
9. SWB	.337***	.323***	.117	.448***	.144	.550***	-.623***	-.644***	.69

*Notes*: Pearson correlation matrix with the variables from Study 2–Sample 1. Williams’ t = t-value results (df = 141) of the pairwise comparison of the correlation coefficients of the ER11 and ER89 scales, ER89 = original version of the ER scale, ER11 = our version of the ER scale, AEW = Active Engagement with the World, RPSS = Repertoire of Problem Solving Strategies, IPS = Integrated Performance under Stress, R-CD-RISC = refined version of the CD-RISC, SWB = Subjective Well-Being

*** p ≤ .001

** *p* ≤ .01

* *p* ≤ .05

**Table 6:** Inter-correlation matrix for Study 2–Sample 2 and comparison of the correlations of the ER11 and ER89 scales.

	1.	2.	3.	4.	5.	6.	7.	8.	9.	10.	11.	12.	13.	14.	15.	16.	17.	18.	Williams’ t
1. ER89																			
2. ER11	.978***																		
3. AEW	.825***	.849***																	-3.46***
4. RPSS	.789***	.799***	.474***																-1.36
5. IPS	.557***	.571***	.268***	.289***															-1.47
6. R-CD-RISC	.656***	.651***	.403***	.602***	.533***														.47
7. Positive Affect (moment)	.447***	.435***	.256***	.526***	.190***	.464***													1.19
8. Positive Affext (week)	.462***	.455***	.296***	.544***	.153**	.564***	.619***												.70
9. Negative Affect (moment)	-.161**	-.139*	-.020	-.155**	-.201***	-.349***	-.187***	-.205***											-1.86
10. Negative Affect (week)	-.243***	-.232***	-.070	-.230***	-.308***	-.445***	-.293***	-.332***	.712***										-.97
11. Extraversion	.569***	.582***	.393***	.646***	.247***	.528***	.402***	.490***	-.176**	-.267***									-1.38
12. Agreeableness	.395***	.318***	.198***	.302***	.247***	.317***	.298***	.289***	-.251***	-.326***	.382***								7.59***
13. Conscientiousness	.342***	.304***	.116*	.470***	.096	.407-**	.366***	.412***	-.211***	-.300***	.359***	.217***							3.48***
14. Emotional Stability	.474***	.461***	.205***	.403***	.568***	.645***	.355***	.386***	-.522***	-.644***	.362***	.483***	.321***						1.24
15. Openness	.581***	.571***	.581***	.453***	.146**	.338***	.322***	.300***	-.009	-.046	.334***	.212***	.134*	.154**					1.03
16. Stability	.542***	.487***	.233***	.519***	.422***	.617***	.452***	.482***	-.448***	-.577***	.489***	.755***	.663***	.826***	.221***				5.74***
17. Plasticity	.703***	.706***	.592***	.677***	.243***	.534***	.445***	.488**-	-.117*	-.196***	.830***	.367***	.307***	.320***	.803***	.440***			-.30
18. GSE	.530**	.520***	.310***	.604***	.258***	.728***	.426***	.561***	-.223***	-.317***	.479***	.281***	.562***	.433***	.308***	.563***	.485***		1.05
19. BICB	.346**	.349***	.302***	.352***	.075	.266***	.281***	.310***	-.023	-.026	.261***	.043	.184***	.129*	.362***	.157**	.379***	,291***	-.27

Pearson correlation matrix with the variables from Study 2—Sample 2. Williams’ t = t-value results (df = 318) of the pairwise comparison of the correlation coefficients of the ER11 and ER89 scales, ER89 = original version of the ER scale, ER11 = our version of the ER scale, AEW = Active Engagement with the World, RPSS = Repertoire of Problem Solving Strategies, IPS = Integrated Performance under Stress, R-CD-RISC = refined version of the CD-RISC, PA (moment) = positive affect measured by PANAS based on the respondents’ actual state, PA (week) = positive affect measured by PANAS based on the last week of the respondents, NA (moment) = negative affect measured by PANAS based on the respondents’ actual state, NA (week) = negative affect measured by PANAS based on the last week of the respondents, BICB = Biographical Inventory of Creative Behaviors

*** p ≤ .001

** *p* ≤ .01

* *p* ≤ .05

The hierarchical multiple regression models of ER11 and R-CD-RISC (see Table 7) showed that ER11 did not contribute to the explained variance if it was included in second step of the analyses or it lost its significant effect after R-CD-RISC was included in the model in case of State Anxiety, Subjective Well-Being, Negative Affect (last week), General Self-Efficacy, and the Big Five traits of Agreeableness and Conscientiousness. In some models, both predictors contributed to the explained variance, but R-CD-RISC had a greater coefficient in the case of Trait Anxiety, the remaining three PANAS scales, and the Stability meta-trait than ER11. ER11 had higher regression coefficients than R-CD-RISC in the case of the Extraversion trait and the Plasticity meta-trait of the Big Five. R-CD-RISC did not contribute to the explained variance if it was included in step two or it lost its significant effect after ER11 was included in the model in the case of Openness and creativity measured by BICB. In the case of Agreeableness, the contribution from the two variables was almost identical.

**Table 7:** Incremental validity testing of the ER11 through hierarchical multiple linear regressions.

	Predictors	*F*_shared_	R^2^_shared_	R^2^_individual_	R^2^_change_	*F*_change_	*t*-value	*β*
*Sample 1* (N = 144, df = 1,143)								
1. Trait Anxiety	ER11	69.57***	49.7% (49.0%)	25.2%	1.4%	3.97*	-1.99*	-.146
	R-CD-RISC			48.3%	24.5%	68.65***	-8.29***	-.609
2. State Anxiety	ER11	23.15***	24.7% (23.7%)	7.5%	0.0%	.08	.27	.025
	R-CD-RISC			24.7%	17.2%	32.30***	-5.68***	-.511
3. Subjective Well-being	ER11	31.42***	.30.8% (29.8%)	9.7%	0.0%	.05	-.22	-.019
	R-CD-RISC			30.8%	21.1%	43.11***	6.57***	.566
*Sample 2* (N = 321, df = 1,320)								
PANAS								
4. Positive Affect (moment)	ER11	51.84***	24.6% (24.1%)	18.9%	3.1%	12.89***	3.59***	.230
	R-CD-RISC			21.5%	5.7%	23.93***	4.89***	.314
5. Positive Affect (week)	ER11	78.67***	33.1% (32.7%)	20.7%	1.3%	6.32*	2.51*	.152
	R-CD-RISC			31.8%	12.4%	59.09***	7.69***	.465
6. Negative Affect (moment)	ER11	24.95***	13.6% (13.0%)	1.9%	1.4%	4.97%	2.23*	.153
	R-CD-RISC			12.2%	11.6%	42.74***	-6.54***	-.449
7. Negative Affect (week)	ER11	40.60***	20.3% (19.8%)	5.4%	0.6%	2.31	1.52	.100
	R-CD-RISC			19.8%	15.0%	59.75***	-7.73***	-.510
BFI								
8. Extraversion	ER11	96.34***	37.7% (37.3%)	33.9%	9.9%	50.44***	7.10***	.414
	R-CD-RISC			27.9%	3.8%	19.56***	4.42***	.258
9. Agreeableness	ER11	22.15***	12.2% (11.7%)	10.1%	2.2%	7.88**	2.81**	.194
	R-CD-RISC			10.1%	2.1%	7.57**	2.75**	.190
10. Conscientiousness	ER11	32.15***	16.8% (16.3%)	9.3%	0.3%	1.03	1.01	.068
	R-CD-RISC			16.6%	7.6%	28.90***	5.38***	.362
11. Emotional Stability	ER11	114.38***	41.8% (41.5%)	21.3%	0.3%	1.62	1.27	.072
	R-CD-RISC			41.5%	20.6%	112.47***	10.61***	.598
12. Openness	ER11	77.58***	32.8% (32.4%)	32.6%	21.4%	101.14***	10.06***	.609
	R-CD-RISC			11.4%	0.2%	.95	-.973	-.059
13. Stability	ER11	103.10***	39.3% (39.0%)	23.7%	1.2%	6.52**	2.55**	.147
	R-CD-RISC			38.1%	15.7%	82.08***	9.06***	.521
14. Plasticity	ER11	164.08***	50.8% (50.5%)	49.8%	22.3%	144.09***	12.00***	.622
	R-CD-RISC			28.5%	0.9%	6.13*	2.48*	.128
15. General Self-Efficacy	ER11	181.64***	53.3% (53.0%)	27.0%	0.4%	2.51	1.58	.080
	R-CD-RISC			53.0%	26.3%	179.05***	13.38***	.676
16. BICB	ER11	22.55***	12.4% (11.9%)	12.2%	5.3%	19.40***	4.40***	.305
	R-CD-RISC			7.1%	0.3%	.956	.978	.068

df = Degrees of freedom, *F*_shared_ = F-value of the multiple regression model with significance levels, R^2^_shared_ = Explained variance of the model with both predictors are included, Adjusted R-squared in parenthesis, R^2^_individual_ = Explained variance of model if the predictor is included in Step One, R^2^_change_ = Change of explained variance of the model if the predictor is included in Step Two, *F*_change_ = *F*-value change if the predictor is included in step two with significance levels, *t*-value = *t*-value statistic of the predictor if both predictors are included with significance levels, β = Standardized beta coefficient of the predictor, ER11 = our version of the ER scale, R-CD-RISC = Refined Connor-Davidson Resiliency Scale, PANAS = Positive and Negative Affect Scales, BFI = Big Five Inventory, BICD = Biographical Inventory of Creative Behaviors

***p < .001

**p < .01

*p < .05

It seems that whenever the component model was significant, RPSS played a very important role because it was always a significant predictor (Table 8). It was the only significant predictor in the case of Subjective Well-Being, General Self-Efficacy, and Positive Affects measured by PANAS. IPS and RPSS were the significant predictors in those models which included variables related to negative emotions, namely Negative Affects, Trait and State Anxiety, Agreeableness, Emotional Stability, and the meta-trait Stability. AEW and RPSS were significant predictors in the case of Extraversion, Conscientiousness, Openness, the meta-trait Plasticity, and everyday creativity. Surprisingly, AEW related negatively to Conscientiousness, while RPSS was related positively to it. Please note, that those models in which AEW and RPSS appeared together as significant predictors were the ones in which ER11 had a greater coefficient than R-CD-RISC or R-CD-RISC did not contribute to the explained variance in a significant way.

**Table 8:** Construct validity testing of the dimensions of the ER11 through multiple linear regressions.

	Sample 1 (*N* = 144, df = 1,143)			Sample 2 (*N* = 321, df = 1,320)												
	Trait Anxiety	State Anxiety	SWB	PA (mom)	PA (week)	NA (mom)	NA (week)	Extra	Agree	Consci	EmStab	Open	Stability	Plasticity	GSE	BICB
R^2^	45.6% (44.4%)	16.7% (15.0%)	19.6% (17.8%)	27.8% (27.1%)	29.8% (29.1%)	5.8% (5.0%)	12.3% (11.5%)	43.0% (42.4%)	12.0% (11.2%)	23.6% (22.8%)	38.8% (38.3%)	38.1% (37.5%)	35.3% (34.7%)	55.3% (54.9%)	37.2% (36.6%)	15.0% (14.2%)
*F*	39.08***	9.39***	11.35***	40.64***	44.81***	6.56***	14.87***	79.64***	14.45***	32.59***	67.09***	65.06***	57.80***	130.8***	62.68***	18.67***
	*β*	*β*	*β*	*β*	*β*	*β*	*β*	*β*	*β*	*β*	*β*	*β*	*β*	*β*	*β*	*β*
**AEW**	-0.043	0.071	0.01	0.002	0.051	0.101	0.097	.103*	0.043	-.133*	-0.067	.482***	-0.069	.350***	0.016	.183**
**RPSS**	-.440***	-.293***	.429***	.513***	.523***	-.150*	-.196***	.583***	.232***	.540***	.289***	.240***	.463***	.511***	.571***	.281***
**IPS**	-.417***	-.231**	0.036	0.042	-0.012	-.185***	-.277***	0.051	.169**	-0.025	.503***	-0.052	.306***	0.001	0.088	-0.055

df = Degrees of freedom, *F* = F-value of the multiple regression model with significance levels, R^2^ = Explained variance of the model, Adjusted R-squared in parenthesis, β = Standardized beta coefficient of the predictor, AEWW = active engagement with the world, RPSS = repertoire of problem solving strategies, SWB = Subjective Well-Being Scale, PA (mom) = Positive affect dimension of the PANAS refereeing to the actual emotional state of the respondent, PA (week) = Positive affect dimension of the PANAS refereeing to the emotional state of the respondent over the last week, NA (mom) = Negative affect dimension of the PANAS refereeing to the actual emotional state of the respondent, NA (week) = Negative affect dimension of the PANAS refereeing to the emotional state of the respondent over the last week, Extra = Extraversion trait of the BFI, Agree = Agreeableness trait of the BFI, Consci = Conscientiousness trait of the BFI, EmStab = Emotional Stability trait of the BFI, Open = Openness trait of the BFI, GSE = General Self-Efficacy, BICD = Biographical Inventory of Creative Behaviors.

*p < .05

**p < .0,

***p < .001

### Discussion

The validity of the original, 14-item version of the ER89 scale was previously mainly tested with regard to its correlations with other scales [2,3]. In order to assess the convergent and divergent validity of the component model we followed this practice using a comparable strategy. First, we tested the convergent validity of ER11 and ER89. Both scales correlated with other variables in the same direction with very similar magnitudes. However, the comparison of their correlation coefficients revealed that on the one hand, ER89 has a stronger relationship with Trait Anxiety, the Agreeableness and Conscientiousness traits, and the Stability meta-trait of the Big Five, compared to ER11. ER89 was also more strongly connected to R-CD-RISC than th ER11, but only in Sample 1. On the other hand, ER11 was more related to its AEW dimension. In other words, while the two constructs’ relationship pattern concerning other constructs was almost identical, some differences were found. The reason for these differences can be derived from the excluded items. Considering their content, item one (“I am generous with my friends”) and item 9 (“Most of the people I meet are likeable”) are related to Agreeableness, while item ten (“I usually think carefully about something before acting”) is related to Conscientiousness.

Second, we tested the incremental validity of ER11 compared to R-CD-RISC [25]. R-CD-RISC showed psychometric superiority over ER11 in the case of State Anxiety, Subjective-Well Being, Negative Affect based on the respondent's last week, General Self-Efficacy, and the Agreeableness and Conscientiousness traits of the Big Five. It explained more variance than ER11 in the case of Trait Anxiety, the PANAS scales, and the Stability meta-trait. These results suggest that R-CD-RISC is a better measure of resilience than ER11. These implications are not surprising as R-CD-RISC was designed carefully to measure positive adaptation in the face of stress or trauma, and self-confidence in one’s ability to overcome these difficulties in life.

Block, on the other hand, conceptualized ER in a broader sense, as a measure of behavioral flexibility. Of course, a behaviorally more flexible individual can also overcome such difficulties. However, according to Block [1], ER is a meta-trait, and it has additional general flexibility of function. Our results confirm this position, as ER11 proved to be better than R-CD-RISC in predicting the scores on Extraversion, Openness, the Plasticity meta-trait, and everyday creativity. We suggest that these conclusions can understood in terms of Lewin’s [4,5] concept of Elasticity and Permeability, or the Plasticity and Stability meta-traits of the Big Five [55–58]. In this framework, the function measured is closer to stability or permeability, serving the need to keep the personality system intact from external threats. The function measured by ER is more related to plasticity or elasticity, providing the ability to flexibly adjust the personality system when necessary. In other words it represents the ability to adapt to changing environmental demands.

This two-faced nature of resiliency can be seen also in the relationship pattern of the three dimensions of ER. Our third aim in Study 2 was to examine how these dimensions are related to other constructs. It turned out that RPSS was a significant predictor in every model, suggesting that in order to behave adaptively it is necessary to possess the skills required to deal with the situation. The second implication of these results is that in every case in which ER11 proved to be a better instrument than R-CD-RISC, both the AEW and RPSS dimension were significant predictors. In cases where negative emotions were included in the construct, such as Negative Affects, both the IPS and RPSS dimensions were significant predictors. These results imply that the permeability or stability function is present within ER through the coupling of IPS and RPSS, while the elasticity or plasticity function is present within ER through the coupling of AEW and RPSS.

It is important to note that despite their differences, ER11 and R-CD-RISC have many similarities. Based on these we suggest that both constructs are able to measure both sides of resiliency, but R-CD-RISC and probably the other measures of resilience are more able to grasp the permeability or stability function, while ER11 is better suited to measuring the elasticity or plasticity function.

To achieve appropriate psychometric properties, three items were excluded from the ER89 scale. As pointed out above, these items are connected to Agreeableness and Conscientiousness. It is possible that the permeability or stability function is better represented by the original scale than by ER11. One could argue that these exclusions weaken the measure, compared to R-CD-RISC. However, in Study 1 it turned out that ER89 did not have the psychometric properties expected from a scale in today’s personality research. Because of this we argue that the best way to improve the scale is not by turning back to the original construct, but by improving the new one by generating more items.

Finally, we would like to call attention to a result which is some way beyond the scope of the current paper, namely that the correlation between the General Self-Efficacy and R-CD-RISC is so high that the independence of the two constructs must be questioned. Previous results also found high correlation between the two constructs [60]. A possible reason for these findings could be that both have items focusing on self-confidence in dealing with adversities in life. The main difference is that the items in R-CD-RISC are more specific than the ones in the New General Self-Efficacy Scale [59]. Based on this it is possible that R-CD-RISC measures a specific self-efficacy focusing on self-efficacy in stressful or traumatic situations. This topic is beyond the scope of this paper, however it might be a fruitful research topic to investigate the different and similar facets of resilience measured by R-CD-RISC and alternative forms of self-efficacy.

## Study 3

In the previous studies, we tested the structural, convergent, and divergent reliability of the refined ER11 scale. However, our samples mainly included university students and young adults. In order to provide information about the more general applicability of this scale we administered it to a representative Hungarian sample. Both Block and Kremen [2] and Letzring *et al*. [3] investigated ER separately for the two genders and revealed some differences between ER in males and females. It seems that males with high ER can be described more by cognitive and intellectual dimensions than females with high ER, while the latter can be characterized more by social dimensions [2]. Finding similar results with the California Q-sort Inventory used in the previously mentioned study, Letzring *et al*. [3] found that ER was a better correlate of subjective well-being in females than in males. Also, adolescent girls with lower ER reported more drug usage than those with higher ER, but this effect was not present among boys [68]. While in the previous studies our aim was to identify the factor structure of ER11 without taking into account potential gender differences, in this study having a representative sample, and thus a balanced gender distribution, we tested the factor structure separately for males and females. Although the ER89-R model did not fit our data in Study 1, we wanted to explore this question using a Hungarian representative sample, as well.

### Methods

#### Participants

604 respondents (51.2% female), aged between 18 and 60 years (*M* = 38.98, *SD* = 12.51) filled out the questionnaires. Regarding place of residence, 117 (19.3%) respondents live in the capital, 120 (19.8%) live in county towns, 179 (29.6%) live in other towns, and 189 (31.2%) live in villages. Regarding level of education, 70 (11.6%) respondents have only primary school education, 384 (63.5%) have secondary level education, 150 (24.8%) have higher level education and one respondent (.2%) did not report the level of education. We conducted CFAs on the basis of the same protocol described in Study 1. Data can be accessed from http://real.mtak.hu/10689/.

#### Measures and Data gathering

Respondents filled out the ER89 questionnaire in an online form among other scales. This sample was representative of the Hungarian population in terms of gender, age, place of residence, and level of education on the basis of the Hungarian Central Statistical Office. This research employed a nationally representative probability sample selected randomly from an Internet-enabled panel including 80000 members with the help of the SolidData LTD in June 2013. For the preparation of the sample a multiple-step, proportionally stratified, probabilistic sampling method was employed. Members of this panel use the internet at least once a week. The panel demography is permanently filtered; therefore, individuals who give responses too quickly, without paying attention to their response, and give fake or unused e-mail addresses are filtered out. The sample was representative of those Hungarians who use the internet at least once a week.

#### Data analysis

We conducted CFAs on the basis of the same protocol described in Study 1. The measurement invariance [69,70] between males and females was analyzed. In the *configural* or baseline setting all values could differ between the groups. In the *weak* invariance setting, the factor loadings were set as invariant. In the *strong* invariance model the factor loadings and the thresholds, whereas in the *strict* invariance the factor loadings, thresholds, and error variances were set to be equal between the groups. Finally, *factor invariance*, in which factor variances were also fixed between groups, was tested. The different nested models were tested with the DIFFTEST procedure of the Mplus software. Student’s *t*-test was used to test any significant differences between genders. The effect sizes for these differences were measured by Cohen’s *d*.

### Results

We tested the factor structure of the scale with confirmatory factor analysis (Table 9.). The original construct of ER and ER89-R did not have a good fit. The 3-factor hierarchical model had a good fit in the overall sample and in males in terms of CFI and TLI, but the RMSEA was above the point of good fit in both models. In females, the CFI and TLI values showed borderline fit, and the RMSEA also showed less acceptable values. The reliability of the overall scale and the subscales was acceptable.

**Table 9:** Structural validity of the ER11 on the representative sample and separately for male and female respondents.

Model	χ2	χ2/DF	CFI	TLI	RMSEA
*All participants* (*N* = 604)					
ER89	668.02	8.68	.872	.849	.113
ER89-R	253.98	7.47	.909	.879	.103
ER11 (first-order)	190.12	4.64	.964	.951	.078
*Males* (*N* = 295)					
ER89	333.60	3.94	.898	.880	.106
ER11 (first-order)	124.29	3.03	.963	.951	.083
*Females* (*N* = 309)					
ER89	452.48	5.58	.830	.799	.126
ER11 (first-order)	145.66	3.55	.947	.928	.091

*Notes*: χ^2^ = Chi-square, χ2DF = Chi-square / Degree of freedom ratio; CFI = comparative fit index, TLI = Tucker-Lewis index, RMSEA = root mean square of approximation.

All measurement models fitted the data similarly to the single group CFAs (Table 10). The weak invariance model (Model 2) did not differ from the configural model (Model 1) significantly, thus metric invariance was established. This suggests that the latent factors have the same meaning across the groups. The difference between the weak (Model 2) and strong measurement models (Model 3) was significant, thus scalar non-invariance was established. Modification indices revealed that the main source of this difference was threshold three of item 6. While the difference between the weak (Model 2) and the partial strong model (Model 4) decreased, their difference was still significant. This indicates that the groups have different means on the factors. Please note that the difference between the two CFI and RMSEA indices are slight. This suggests that the scalar non-invariance might not be truly pronounced.

**Table 10:** Summary for the goodness of fit statistics for models on the measurement invariance testing of gender.

	χ^2^	χ^2^/DF	CFI	RMSEA	Comp.	Δχ^2^	ΔCFI	ΔRMSEA
Model 1–configural	270.41	3.30	.956	.087				
Model 2–weak	247.95	2.75	.963	.076	Model 1	15.13	.007	.011
Model 3–strong	304.62	2.54	.956	.071	Model 2	65.48***	.007	.005
Model 4–strong partial	294.42	2.47	.959	.070	Model 2	54.72**	.004	.001
Model 5–strict	271.45	2.51	.961	.071	Model 4	34.38***	.002	.001
Model 6–strict partial	288.17	2.44	.960	.069	Model 4	7.17**	.001	.001
Model 7–factor	251.35	2.08	.969	.060	Model 6	2.50	.009	.009

configural invariance = all values are free, weak invariance = factor loadings are invariant, strong invariance = factor loadings and intercepts are invariant, strict invariance = factor loadings, intercepts, and error variances are invariant, χ^2^ = Chi-square, χ2DF = Chi-square / Degree of freedom ratio; CFI = comparative fit index, TLI = Tucker-Lewis index, RMSEA = root mean square of approximation; Comp = to which model the current one is compared to; Δχ2 = chi-square test difference, significance level is indicates; ΔCFI = comparative fit index difference; ΔRMSEA = root mean square error of approximation difference.

*p < .05

**p < .01

***p < .001

The difference between the partial strong (Model 4) and strict invariance models (Model 5) was significant. Modification indices revealed that that the source of this misfit was item 8. After freeing this item, the difference became less prominent between the partial strong (Model 4) and partial strict (Model 6) models, but it was still significant. However, the difference in terms of the RMSEA and CFA was almost non-existent. Based on this the suggestion that group comparisons based on the summation of the scales are not valid was rejected. Finally, the factor invariance model (Model 7) did not differ from the partial strict one (Model 6), thus the variance in each of the factors is similar for both groups.

The descriptive statistics seem to confirm this finding (Table 11). Males differed significantly from females in ER11 (*t*(602) = 2.260, *p* = .024, Cohen’s *d* = .184), AEW (*t*(602) = 2.029, *p* = .043, Cohen’s *d* = .165), and IPS (*t*(602) = 4.744, *p* < .001, Cohen’s *d* = .386), but not in RPSS (*t*(602) = -.193, *p* = .847, Cohen’s *d* = -.016). There were no significant differences between the age groups. The only exception was found in the IPS scale: female age groups differed from each other significantly (*F*(4, 308) = 2.966, *p* = .020). However, the Tukey’s HSD post-hoc test only revealed a tendency (*p* = .060) showing that the 51–60 age group scored higher on this scale than the 31–40 age group. The reliability of ER11 and its three subscales in described in Table 11.

**Table 11:** Descriptive statistics for the ER11 and its subscales.

		ER11	AEW	RPSS	IPS
*All participants*	*M*	2.90	2.90	2.96	2.79
(*N* = 604)	*SD*	.51	.62	.54	.72
	Skewness	-.29	-.39	-.21	-.26
	Kurtosis	.57	-.04	.33	-.25
	α	.844	.778	.699	.672
	ω	.880	.801	.723	.692
	ω_h_	.791	.259	.000	.310
*Males*	*M*	2.95	2.95	2.96	2.93
(*N* = 295)	*SD*	.51	.61	.54	.68
	Skewness	-.26	-.36	-.13	-.44
	Kurtosis	.76	.09	.35	.18
	α	.861	.789	.728	.602
	ω	,879	.799	.735	.619
	ω_h_	.802	.243	.015	.173
*Females*	*M*	2.86	2.85	2.96	2.66
(*N* = 309)	*SD*	.50	.63	.54	.72
	Skewness	-.33	-.42	-.28	-.09
	Kurtosis	.40	-.16	.34	-.38
	α	.827	.766	.676	.711
	ω	.878	.798	.720	.726
	ω_h_	.776	.278	.000	.388

*Notes*: α = Cronbach’s alpha

### Discussion

Results of the online representative sample suggest that ER11 has acceptable internal consistency, but a less acceptable structural validity on the more diverse sample than used in Study 1. As mentioned in Study 2 we believe that the next step to improve the scale so that it can measure the ER meta-trait in a more reliable way is to generate more items and include them in the scale instead of turning back to the original ER89 scale.

While there were no gender differences in terms of model fit, measurement invariance testing suggested that although both genders share the factor structure of ER, the means associated with the latent variables are different; males generally have higher ER than females. The largest gender difference was found in IPS. This result is in line with that of Block and Kremen [2] who claimed that resilient males have to learn and practice more how to control their aggressive impulses than females and IPS measures a quick recovery from anger and stress. A possible limitation is that based only on the Chi-square differences of the strong and strict invariance models, males and females could not be compared to each other based on summed item scores. However, the difference between the models in terms of other modification indices were extremely low, thus this interpretation can be rejected.

## General Discussion

The purpose of the present research was to identify the components of ego-resiliency and to provide insights into the mechanisms of flexibility and resilience in the face of the constantly changing environmental demands. In a series of studies we identified three components of Block’s [1] ego-resiliency. These components are distinct from each other based on their factor structure (Study 1 and 3), and they have their own, distinguishable relationship pattern with other constructs (Study 2).

These relationship patterns suggest that despite being embedded in a general personality dimension, the components are responsible for different functions of ER. One function can be described as stability or resilience and keeps the personality system intact when it is faced with distress or other harmful effects. The other function can be construed as plasticity or flexibility, responsible for adaptively changing the personality system when it is necessary because of internal or environmental demands.

These functions are manifested by the interplay between the three components of ER. *Active Engagement with the World* (AEW) reflects the open-minded experience and information seeking tendency of the individual. *Repertoire of Problem Solving Strategies* (RPSS) incorporates a broad set of skills in the social, personal, and cognitive domains of personality and the positive interpretation of life events reflected in higher subjective well-being; i.e. skills required to provide a well-fitting response to the actual situation. *Integrated Performance under Stress* (IPS) describes the tendency for fast and effective coping with stressful events or threats. If the personality system is in a situation where it has to protect itself from harm, IPS and RPSS together make up the stability or resilience function of ER. However, when it is faced with a situation where plasticity or adaptation is required, ER can still provide that function through the coupling of AEW and RPSS. This means that ER can be described as a *general resiliency* of personality, because both functions are integrated into it.

While the theories of resilience [24] incorporate the notion of plasticity and adaptivity, they put their emphasis on the stability and conservation-related function of resiliency. The focus is on the properties of the personality that make the individual resilient to adversities, traumas, and stress. The scales that measure resilience, like R-CD-RISC [25] accomplish their task better in terms of this function than the ER89 or ER11 scales. However, ER89 and ER11 are better suited to measuring the plasticity or flexibility-related functions of resiliency.

In his theory, Block [1] conceived personality as an affect processing system. The mechanism of ego-resiliency (thus the general flexibility of behavior) is modulated by anxiety: internal or external causes produce anxiety which triggers the function of ER. ER modifies the level of control adaptively, so that accommodation or assimilation is selected as a strategy. In terms of the model of general resiliency, adaptive behavior can be described as the following: IPS starts the process by informing the system about anxiety. Then, through the function of the other two components assimilation or accommodation is chosen. This contradicts our present results, as IPS was not a significant predictor in many cases, while AEW and RPSS were.

A possible solution to this contradiction might come from understanding that Block used the permeability and elasticity functions of Lewin. He formulated ego-control to measure the permeability of the personality system, and ER to measure the elasticity. From the present results it seems that ER on its own incorporates both of these functions. However, further testing is required to measure how the stability aspect of ER is connected to ego-control.

The present results and the model of general resiliency call attention to the dynamic plasticity of behavior and personality, and the dynamic interactions between the individual and the surrounding environment, which is relatively neglected in personality psychology. This has always been a complicated field to study (see [71]), but this scale could be a useful tool for understanding this phenomenon. The perspective of embodied cognition (see [72]) could provide a good basis for this kind of research. However, more direct testing of the assumed processes underlying the components are crucial, possibly using behavioral methods.

Moreover, based on Study 3, males and females have a different emphasis on the components and the ER itself. In the history of ER, Block always put an emphasis on the examination of males and females separately (e.g. [2,3]). Further examination of gender differences in the proposed framework could lead to a better understanding of resiliency and flexibility as expressed in males and females.

An important limitation of the current study is that all respondents were Hungarian; it would be useful to replicate these results in other cultures. While the hierarchical factor-solution of ER showed a good fit in all three samples of Study 1, its fit was borderline in the representative sample of Study 3. Furthermore, except for the test-retest reliability assessment, the present studies used cross-sectional measurements. However, it would be important to examine the course of the ER components longitudinally, and to collect more behavioral data on how the level of general resiliency and its components change in different contexts.

The final scale is only 11 items long, and we believe that it would benefit from additional items. As a follow on, we intend to generate more items in accordance with the proposed theoretical framework to improve the scale. This way the new version of ER11 could measure the stability function of resiliency as well as the modern resilience scales (e.g. R-CD-RISC). Finally, it would be fruitful to examine how components of ER can be improved by training targeted at the adaptive adjustment of ego-control.

Applied personality research could benefit from this model of general resiliency, because the function of plasticity can be informative regarding adaptive problem solving in various everyday situations, even if they lack stress and trauma but still require flexibility. Many jobs, for example, are rarely traumatic, but they require flexible adaptation to various situations in terms of active engagement with the world, drawing on one’s repertoire of problem solving strategies to accomplish the tasks and to solve the work-related problems. Applied research focusing on the plasticity function of ER could benefit from using this construct in several fields, including e.g. tutoring or coaching. Moreover, items of the scale are not transparent, that is they are quite oblique about what they measure exactly. This feature of the scale would make it a useful tool in selecting candidates for work positions which require flexibility.

## Conclusion

In a series of studies we identified and tested the components that form Block and Block’s [6] ego-resiliency. With factor analytical methods we distinguished three components of ER and named them in accordance with Block and Block’s [6] assumption regarding the building blocks of their meta-trait: active engagement with the world (AEW), repertoire of problem solving strategies (RPSS) and integrated performance under stress (IPS). Results show that with these components two important functions of resiliency, namely resilience or stability and flexibility or plasticity can be distinguished from each other. These dimensions are highly compatible with the meta-traits identified by DeYoung *et al*. [55–58] which integrate the Big Five traits. While both constructs measure both functions, theories of resilience (R-CD-RISC) emphasize stability, while ER is better suited to measuring plasticity. Block [1] emphasized a personality system which can be elastic and permeable. He proposed two personality constructs, EC and ER, however the present results suggest that ER incorporates both functions. Block also conceived personality as an affect processing system, which can provide adaptive response in face of anxiety. In our proposed model of general flexibility, the plasticity or elasticity functions provide adaptive behavior also in situations without a significant amount of stress. The present results suggest that the identification of the building blocks of ER could extend the scope of both Block’s concept and theories of resilience, and may lead to a new theoretical framework involving a meta-trait of general flexibility.

## Supporting Information

S1 File Factors and Items of Ego-Resiliency Scale. (DOCX)

## References

[1] Block J. Personality as an affect-processing system. Mahwah, NJ: Erlbaum; 2002.

[2] Block JH, Kremen AM. IQ and ego-resiliency: Conceptual and empirical connections and separateness. J Pers Soc Psychol. 1996; 70: 349–361. 10.1037/0022-3514.70.2.349 8636887

[3] Letzring TD, Block J, Funder DC. Ego-control and ego-resiliency: Generalization of self-report scales based on personality descriptions from acquaintances, clinicians, and the self. J Res Pers. 2005; 39: 395–422. 10.1016/j.jrp.2004.06.003

[4] Lewin K. Principles of topological psychology New York: McGraw-Hill; 1936.

[5] Lewin K. Field theory in social science. New York: Harper; 1951.

[6] Block JH, Block J. The role of ego-control and ego-resiliency in the organization of behavior In: Collins WA, editor. Development of cognition, affect, and social relations: The Minnesota symposia on child psychology. Hillsdale, NJ: Erlbaum; 1980 pp. 39–101.

[7] Funder DC, Block JH, Block J. Delay of gratification: Some longitudinal personality correlates. J Pers Soc Psychol. 1983; 44: 1198–1213. 10.1037/0022-3514.44.6.1198

[8] Tugade MM, Fredrickson BL. Resilient individuals use positive emotions to bounce back from negative emotional experiences. J Pers Soc Psychol. 2004; 86: 320–333. 10.1037/0022-3514.86.2.320 14769087 PMC3132556

[9] Caldwell JG, Shaver PR. Exploring the cognitive-emotional pathways between adult attachment and ego-resiliency. Individual Differences Research. 2012; 10: 141–152.

[10] Paulhus DL, Martin CL. The structure of personality capabilities. J Pers Soc Psychol. 1987; 52: 354–365. 10.1037/0022-3514.52.2.354

[11] Colvin CR, Block J, Funder DC. Overly positive self-evaluations and personality: Negative implications for mental health. J Pers Soc Psychol. 1995; 68: 1152–1162. 10.1037/0022-3514.68.6.1152 7608859

[12] Mischel W, Shoda Y, Peake PK. The nature of adolescent competencies predicted by preschool delay of gratification. J Pers Soc Psychol. 1988; 54: 687–696. 10.1037/0022-3514.54.4.687 3367285

[13] Block J, Block JH. Nursery school personality and political orientation two decades later. J Res Pers. 2006; 40: 734–749. 10.1016/j.jrp.2005.09.005

[14] Taylor Z, Sulik M, Eisenberg N, Spinrad T, Silva KM, Lemery-Chalfant K, et al Development of ego-resiliency: Relations to observed parenting and polymorphisms in the serotonin transporter gene during early childhood. Soc Dev. 2013; 23: 433–450. 10.1111/sode.12041 PMC4206910 25346579

[15] Howes C, Matheson CC, Hamilton CE. Maternal, teacher, and child care history correlates of children's relationships with peers. Child Dev. 1994; 65: 264–273. 10.1111/j.1467-8624.1994.tb00749.x 8131652

[16] Taylor ZE, Eisenberg N, Spinrad TL, Eggum ND, Sulik MJ. The relations of ego-resiliency and emotion socialization to the development of empathy and prosocial behavior across early childhood. Emotion. 2013; 15: 822–831. 10.1037/a0032894 PMC4314208 24098930

[17] Taylor ZE, Doane LD, Eisenberg N. Transitioning from high school to college: Relations of social support, ego-resiliency, and maladjustment during emerging adulthood. Emerging Adulthood. 2013: 1–11. 10.1177/2167696813506885

[18] Alessandri G, Vecchione M, Steca P, Caprara MG, Caprara GV. A revised version of Kremen and Block's Ego-Resiliency Scale in an Italian Sample. Testing, Psychometrics, Methodology in Applied Psychology. 2008; 14: 1–19.

[19] Vecchione M, Alessandri G, Barbaranelli C, Gerbino M. Stability and change of ego resiliency from late adoles- cence to young adulthood: A multiperspective study using the ER89-R Scale. J Pers Assess. 2010; 92: 1–10. 10.1080/00223891003670166 20408021

[20] Alessandri G, Vecchione M, Caprara G, Letzring TD. The Ego Resiliency scale revised: A crosscultural study in Italy, Spain, and the United States. Eur J Psychol Assess. 2012; 28: 139–146. 10.1027/1015-5759/a000102

[21] Block J. The challenge of response sets: Unconfounding meaning, acquiescence, and social desirability in the MMPI. New York: Appleton-Century-Crofts; 1965.

[22] Windle G, Bennett KM, Noyes J. A methodological review of resilience measurement scales. Health and Quality of Life Outcomes. 2011; 9: 1–18. 10.1186/1477-7525-9-8 21294858 PMC3042897

[23] Richardson GE. The metatheory of resilience and resiliency. J Clin Psychol. 2002; 58: 307–321. 10.1002/jclp.10020 11836712

[24] Luthar SS, Cicchetti D, Becker B. The construct of resilience: A critical evaluation and guidelines for future work. Child Dev. 2000; 71: 543–562. 10.1111/1467-8624.00164 10953923 PMC1885202

[25] Campell-Sills L, Stein MB. Psychometric analysis and refinement of the Connor–Davidson Resilience Scale (CD-RISC): Validation of a 10-item measure of resilience. J Trauma Stress. 2007; 20: 1019–1028. 10.1002/jts.20271 18157881

[26] Block J. The Q-sort method in personality assessment and psychiatric research. Palo Alto, CA: Consulting Psychologists Press; 1961 10.1007/BF02139257

[27] Khlonen EC. Conceptual analysis and measurement of the construct of ego-resiliency. J Pers Soc Psychol. 1996; 70: 1067–1079. 10.1037/0022-3514.70.5.1067 8656335

[28] Gough HG. Manual for the California Psychological Inventory. Palo Alto, CA: Consulting Psychologists Press; 1957.

[29] Farkas D, Orosz G. The link between ego-resiliency and changes in Big Five traits after decision making: The case of extraversion. Pers Indiv Differ. 2013; 55: 440–445. 10.1016/j.paid.2013.04.003

[30] James G, Witten D, Hastie T, Tibshirani R. An introduction to statistical learning with applications in R. New York: Springer; 2013.

[31] Asparouhov T, Muthén B. Exploratory structural equation modeling. Structural Equation Modeling: A Multidisciplinary Journal. 2009; 16: 397–438. 10.1080/10705510903008204

[32] Tabachnik BG, Fidell LS. Using multivariate statistics. 4th ed ^.^ Boston, MA: Allyn & Bacon; 2001.

[33] Brunner M, Nagy G, Wilhelm O. A tutorial on hierarchically structured constructs. J Pers. 2012; 80: 796–846. 10.1111/j.1467-6494.2011.00749 22091867

[34] Brown TA. Confirmatory factor analysis for applied research. New York: Guilford; 2006.

[35] Schreiber JB, Stage FK, King J, Nora A, Barlow EA. Reporting structural equation modeling and confirmatory factor analysis results: A review. J Educ Res. 2006; 99: 323–337. 10.3200/JOER.99.6.323-338

[36] Hu L, Bentler PM. Cutoff criteria for fit indexes in covariance structure analysis: Conventional criteria versus new alternatives. Structural Equation Modeling: A Multidisciplinary Journal. 1999; 6: 1–55. 10.1080/10705519909540118

[37] Kline RB. Principles and practice of structural equation modeling. New York: Guilford Press; 1998.

[38] Zinbarg RE, Revelle W, Yovel I, Li W. Cronbach’s α, Revelle’s β, and McDonald’s ωH: Their relations with each other and two alternative conceptualizations of reliability. Psychometrika. 2005; 70: 123–133. 10.1007/s11336-003-0974-7

[39] Spielberger CD, Gorsuch RL, Lushene RE. STAI MANUAL for the State-Trait Anxiety Inventory. Palo Alto, CA: Consulting Psychologist Press, Inc; 1970.

[40] Gyollai Á, Simor P, Köteles F, Demetrovics Z. Psychometric properties of the Hungarian version of the original and the short form of the Positive and Negative Affect Schedule (PANAS). Pers Indiv Differ. 2011; 13: 73–79. 21677320

[41] Watson D, Clark LA, Tellegen A. Development and validation of brief measures of positive and negative affect. The PANAS scales. J Pers Soc Psychol. 1988; 54: 1063–1070. 10.1037/0022-3514.54.6.1063 3397865

[42] Diener E, Emmons RA, Larsen RJ, Griffin S. The satisfaction with life scale. J Pers Assess. 1985; 49: 71–75. 10.1207/s15327752jpa4901_13 16367493

[43] Benetti C, Kambouropoulos N. Affect-regulated indirect effects of trait anxiety and trait resilience on self-esteem. Pers Indiv Differ. 2006; 41: 341–352. 10.1016/j.paid.2006.01.015

[44] Giesbrecht T, Abidi K, Smeets T, Merckelbach H, van Oorsouw K, Raymaeker, L. Adversity does not always lead to psychopathology: Cognitive reactivity is related to longitudinal changes in resilience. Netherlands Journal of Psychology. 2009; 65: 62–68. 10.1007/BF03080128

[45] Karaırmak Ö. Establishing the psychometric qualities of the Connor—Davidson Resilience Scale (CD- RISC) using exploratory and confirmatory factor analysis in a trauma survivor sample. Psychiat Res. 2010; 179: 350–356. 10.1016/j.psychres.2009.09.012 20493533

[46] Min JA, Lee NB, Lee CU, Lee C, Chae JH. Low trait anxiety, high resilience, and their interaction as possible predictors for treatment response in patients with depression. J Affect Disorders. 2012; 137 10.1016/j.jad.2011.12.026 22244377

[47] Ong AD, Bergeman CS, Bisconti TL, Wallace KA. Psychological resilience, positive emotions, and successful adaptation to stress in later life. J Pers Soc Psychol. 2006; 9: 730–749. 10.1037/0022-3514.91.4.730 17014296

[48] Singh K, Yu X. Psychometric evaluation of the Connor-Davidson Resilience Scale (CD-RISC) in a sample of indian students. J Psychol. 2010; 1: 23–30.

[49] Steinhardt M, Dolbier C. Evaluation of a resilience intervention to enhance coping strategies and protective factors and decrease symptomatology. J Am Coll Health. 2008; 56: 445–453. 10.3200/JACH.56.44.445-454 18316290

[50] Utsey SO, Nook JN, Fischer N, Belvet B. Cultural orientation, ego resilience, and optimism as predictors of subjective well-being in African Americans. The Journal of Positive Psychology. 2008; 3: 202–210. 10.1080/17439760801999610

[51] Costa P, McCrae R. Revised NEO Personality Inventory (NEO–PI–R) and NEO Five-Factor Inventory (NEO–FFI) professional manual. Odessa, FL: Psychological Assessment Resources; 1992.

[52] John OP, Srivistava S. The Big Five trait taxonomy: History, measurement, and theoretical perspectives In: Pervin LA., John OP, editor. Handbook of personality research: Theory and research. New York: Guilford; 1999 pp. 102–138.

[53] Huey SJ, Weisz JR. Ego control, Ego resiliency, and the Five-Factor Model as predictors of behavioral and emotional problems in clinic-referred children and adolescents. J Abnorm Psychol. 1997; 106: 404–415. 9241942 10.1037//0021-843x.106.3.404

[54] Campell-Sills L, Cohan SL, Stein MB. Relationship of resilience to personality, coping, and psychiatric symptoms in young adults. Behav Res Ther. 2006; 44: 585–599. 10.1016/j.brat.2005.05.001 15998508

[55] DeYoung CG. Higher-order factors of the Big Five in a multi-informant sample. J Pers Soc Psychol. 2006; 91: 11138–11151. 10.1037/0022-3514.91.6.1138 17144770

[56] DeYoung CG, Hasher L, Djikic M, Criger B, Peterson JB. Morning people are stable people: Circadian rhythm and the higher-order factors of the Big Five. Pers Indiv Differ. 2007; 43: 267–276. 10.1016/j.paid.2006.11.030

[57] DeYoung CG, Peterson JB, Higgins DM. Higher-order factors of the Big Five predict conformity: Are there neuroses of health. Pers Indiv Differ. 2002; 33: 533–552. 10.1016/S0191-8869(01)00171-4

[58] DeYoung CG, Peterson JB, Séguin JR, Tremblay RE. Externalizing behavior and the higher order factors of the Big Five. J Abnorm Psychol. 2008; 117: 947–953. 10.1037/a0013742 19025240

[59] Chen G, Gully SM, Eden D. Validation of a New General Self-Efficacy Scale. Organizational Research Methods. 2001; 4: 62–83.

[60] Little LM, Gooty J, Nelson D. Positive psychological capital: Has positivity clouded measurement rigor In: Nelson D, Cooper CL, editors. Positive organizational behavior: Accentuating the positive at work. London: SAGE; 2007 pp. 191–210.

[61] Rutter M. Resilience in the face of adversity: Protective factors and resistance to psychiatric disorder. The British Journal of Psychiatry,. 1985; 147: 598–611. 10.1192/bjp.147.6.598 3830321

[62] Hartley N. Resilience and creativity In: Monroe B, Oliviere D, editors. Resilience in palliative care: Achievement in adversity. Oxford: Oxford University Press; 2007 pp. 281–292.

[63] Batey MD. A psychometric investigation of everyday creativity, University of London 2007.

[64] Silvia PJ, Wigert B, Reiter-Palmon R, Kaufman JC. Assessing creativity with self-report scales: A review and empirical evaluation Psychology of Aesthetics, Creativity, and the Arts. 2011 10.1037/a0024071

[65] Beaton DE, Bombardier C, Guillemin F, Ferraz MB. Guidelines for the process of cross-cultural adaptation of self-report measures. Spine. 2000; 25: 3186–3191. 11124735 10.1097/00007632-200012150-00014

[66] Williams EJ. The comparison of regression variables. J ROY STAT SOC B; 21: 396–399.

[67] Hittner JB, May K, Silver CN. A Monte Carlo evaluation of tests for comparing dependent correlations. J Gen Psychol; 130: 149–168. 10.1080/00221300309601282 12773018

[68] Block J, Block JH, Keyes S. Longitudinally foretelling drug usage in adolescence: Early childhood personality and environmental precursors. Child Dev. 1988; 59: 336–355. 10.2307/1130314 3359859

[69] Marsh HW, Muthén B, Asparouhov T, Lüdtke O, Robizitsch, Morin A, et al Exploratory Structural Equation Modeling, integrating CFA and EFA: Application to students' evaluations of university teaching. Structural Equation Modeling: A Multidisciplinary Journal. 2009; 16: 439–476. 10.1080/10705510903008220

[70] van de Schoot R, Lugtig P, Hox J. A checklist for testing measurement invariance. European Journal of Developmental Psychology. 2012 10.1080/17405629.2012.686740 23024688

[71] Mischel W, Shoda Y. A cognitive-affective system theory of personality: reconceptualizing situations, dispositions, dynamics, and invariance in personality structure. Psychol Rev. 1995; 102 10.1037/0033-295x.102.2.246 7740090

[72] Meier BP, Schnall S, Schwarz N, Bargh JA. Embodiment in social psychology. Topics in Cognitive Science. 2012; 4: 1–12. 10.1111/j.1756-8765.2012.01212.x 22777820

