# Supplementary material for: Ego-Resiliency Reloaded: A Three-Component Model of General Resiliency
Source: PLoS One. 2015 Mar 27;10(3):e0120883. doi: 10.1371/journal.pone.0120883 (PMC4376776; doi:10.1371/journal.pone.0120883)
Supplement: S1 File — (DOCX) [file pone.0120883.s001.docx]

*Active engagement with the world*

3. I enjoy dealing with new and unusual situations.

5. I enjoy trying new foods I have never tasted before.

7. I like to take different paths to familiar places.

8. I am more curious than most people.

11. I like to do new and different things.

*Integrated performance under stress*

2. I quickly get over and recover from being startled.

14. I get over my anger at someone reasonably quickly.

*Repertoire of (social, personal and cognitive) problem solving strategies*

4. I usually succeed in making a favorable impression on people.

6. I am regarded as a very energetic person.

12. My daily life is full of things that keep me interested.

13. I would be willing to describe myself as a pretty ‘‘strong’’ personality.

*Excluded items:*

1. I am generous with my friends.

9. Most of the people I meet are likeable.

10. I usually think carefully about something before acting
